# Unveiling Kaposi Sarcoma: A Case Report on the Clinical Journey in an HIV-Infected Patient

**DOI:** 10.7759/cureus.117473

**Published:** 2026-10-05

**Authors:** Tzipora Levitt, Amy Chen, Jonathan Shadan, Zehava Graber, Ricardo Lopez, Celeste M Defillo-Lopez

**Affiliations:** 1 Medicine, New York Institute of Technology (NYIT) College of Osteopathic Medicine, Old Westbury, USA; 2 Critical Care Medicine, Internal Medicine, and Pulmonary Diseases, Icahn School of Medicine at Mount Sinai, Queens Hospital Center/HHC, New York, USA; 3 Internal Medicine, Icahn School of Medicine at Mount Sinai, Queens Hospital Center/HHC, New York, USA

**Keywords:** aids-related malignancy, hhv-8, hiv/aids, kaposi sarcoma, lower extremity lymphedema, pegylated liposomal doxorubicin, pembrolizumab, pulmonary kaposi sarcoma, tree-in-bud opacities, visceral kaposi sarcoma

## Abstract

Kaposi sarcoma (KS) is an angioproliferative neoplasm associated with human herpesvirus-8 (HHV-8) and remains one of the most prevalent acquired immunodeficiency syndrome (AIDS)-defining malignancies globally. Although its incidence has decreased with the widespread implementation of antiretroviral therapy (ART), KS continues to pose therapeutic challenges, particularly in patients with advanced or recurrent disease. We present the case of a 31-year-old man with human immunodeficiency virus (HIV) on ART who developed recurrent KS with cutaneous and pulmonary involvement. The patient had multiple complaints of progressive bilateral thigh and groin swelling and was treated with pegylated liposomal doxorubicin (PLD). This case underscores the aggressive nature of AIDS-related KS in young patients despite ART and first-line chemotherapy, highlighting the critical importance of early recognition, individualized management, and adherence to treatment protocols to optimize patient outcomes.

## Introduction

Kaposi sarcoma (KS) is a vascular tumor triggered by human herpesvirus-8 (HHV-8), also referred to as KS-associated herpesvirus. This virus is mainly spread through sexual contact, particularly activities that involve the exchange of saliva or genital secretions, but it can also be transmitted through non-sexual saliva exposure and, less commonly, through blood exposure [1]. Once contracted, the virus remains in the body for life, and although most individuals do not show symptoms, KS can develop in those with compromised immune systems, especially in advanced human immunodeficiency virus (HIV) cases [2].

KS typically manifests on the skin and mucous membranes as violaceous patches or nodules, but it can also affect the lymphatic system, lungs, and gastrointestinal tract [2]. When the disease becomes visceral, it can be severe, causing effusions, bleeding, and blockages. Pulmonary lesions can lead to shortness of breath or hemoptysis. Pulmonary involvement has been reported in approximately 6%-32% of patients with acquired immunodeficiency syndrome (AIDS)-related Kaposi sarcoma and is often associated with advanced immunosuppression and concurrent mucocutaneous disease [3]. Gastrointestinal lesions can cause bleeding or obstruction, among other symptoms. Proliferative skin and subcutaneous lesions can compress lymphatic channels, potentially leading to lymphedema [4]. There are four primary types of KS: (1) classical, (2) endemic (African), (3) iatrogenic (transplant-related), and (4) AIDS-related, with the latter being the most aggressive [5]. Prognosis in AIDS-related KS is commonly assessed using the AIDS Clinical Trials Group (ACTG) staging system, which categorizes patients by tumor burden (T), immune status (I), and systemic illness (S) [6].

Although the incidence of KS has declined dramatically with widespread use of antiretroviral therapy (ART), it remains one of the most common AIDS-defining malignancies worldwide, with the incidence highest in untreated or advanced HIV [7]. While ART can improve outcomes, patients with advanced or recurrent KS often need systemic chemotherapy, with pegylated liposomal doxorubicin (PLD) being the recommended first-line systemic therapy [8]. Despite advances in therapy, KS continues to pose diagnostic and therapeutic challenges, particularly in young patients with HIV with recurrent or visceral disease. Here, we present the case of a 31-year-old man with HIV and AIDS-related KS with mucocutaneous and pulmonary involvement, previously treated with PLD, who presented with progressive bilateral thigh swelling and was diagnosed with recurrent disease.

## Case presentation

A 31-year-old man with a medical history of pulmonary embolism (PE), prior syphilis, a self-reported history of skin cancer over the abdominal wall, and human immunodeficiency virus (HIV) complicated by KS presented to the emergency department (ED) with five days of progressive bilateral thigh induration and swelling of the groin. He also reported a one-week history of upper respiratory tract infection symptoms, including fever and a productive cough with tan sputum, which resolved three days prior to presentation. Following the fever, he developed bilateral thigh induration, which was worse on the left, along with dusky discoloration of the groin. The affected area was non-tender, but he described the area as “tight" with movement. The patient denied cough, chest pain, shortness of breath, abdominal pain, nausea, and vomiting. The patient reported being sexually active with men but denied any recent sexual encounters. The patient also denied any history of alcohol, drug, or tobacco use.

The patient was diagnosed with HIV approximately one year prior to the current presentation and was started on ART with dolutegravir, emtricitabine, and tenofovir, as well as Bactrim DS, to which he reported compliance. Shortly after his HIV diagnosis, he developed scattered skin lesions on his back, chest, and nose. He subsequently underwent evaluation with a skin biopsy, which confirmed Kaposi sarcoma. Over the following six months, he completed his first course of pegylated liposomal doxorubicin (PLD). During this time, he was also found to have bilateral pleural effusions and a pulmonary embolism. This prompted further evaluation with bronchoscopy, transbronchial biopsy, and bilateral thoracentesis. Bronchoscopy revealed an intensely violaceous, flat, 8-mm macular lesion, with biopsy findings suggestive of Kaposi sarcoma. Pleural fluid analysis was inflammatory. Prior to starting chemotherapy, his interferon-gamma release assay (IGRA) and sputum smear and culture were negative for acid-fast bacilli (AFB), and subsequent IGRA and sputum testing remained negative.

At the time of his current ED presentation, the patient was afebrile and tachycardic with a heart rate of 127. Significant findings on physical examination included bilateral lower leg pitting edema below the knee, with dark discoloration, without crepitus or fluctuance. He also had swelling in the lower abdomen, scrotal and penile edema, and non-tender, soft, dark papules on his chest, back, and nose. Laboratory testing was notable for a CD4 count of 94 cells/µL and an HIV-1 viral load of 146 copies/mL without resistance markers, along with elevated inflammatory markers, including interleukin-6 (IL-6), erythrocyte sedimentation rate (ESR), and C-reactive protein (CRP). Comprehensive metabolic panel (CMP) showed normal renal and hepatic function, aside from a mildly elevated alkaline phosphatase level of uncertain significance. Additional laboratory findings are summarized in Table 1.

**Table 1:** Summary of key laboratory findings at the time of clinical evaluation, including immunologic, virologic, and inflammatory markers CRP: C-reactive protein, ESR: erythrocyte sedimentation rate, HIV: human immunodeficiency virus, IL-6: interleukin-6 *Reference range varies by age and sex.

Laboratory investigation	Result	Reference range
Leukocyte count	Within normal limits	4-11 × 10³/µL
CD4 count	94 cells/µL	500-1,500 cells/µL
HIV-1 viral load	146 copies/mL	Undetectable (<20-50 copies/mL)
HIV resistance markers	None detected	None detected
Urinalysis (protein)	30 mg/dL	Negative to trace (<10-20 mg/dL)
D-dimer	530 ng/mL	<500 ng/mL (FEU)
IL-6	15.1 pg/mL	<7 pg/mL
ESR	44 mm/hr	<20 mm/hr*
CRP	85.60 mg/L	<3 mg/L

Abdominal ultrasound demonstrated gallbladder sludge and an echogenic liver compatible with nonspecific hepatic parenchymal disease but was otherwise unremarkable. CT of the abdomen and pelvis revealed minor pleural effusions, splenomegaly, mild generalized enlargement of the pancreas, and mesenteric lymphadenitis. Bilateral lower extremity Dopplers were negative for deep vein thrombosis. CT pulmonary angiography was negative for PE; however, it demonstrated bilateral moderate interstitial edema consistent with volume overload, and small pleural effusions more pronounced on the right and at the lung bases. Additional chest CT revealed bilateral interstitial infiltrates with tree-in-bud opacities (Figures 1, 2).

**Figure 1:**
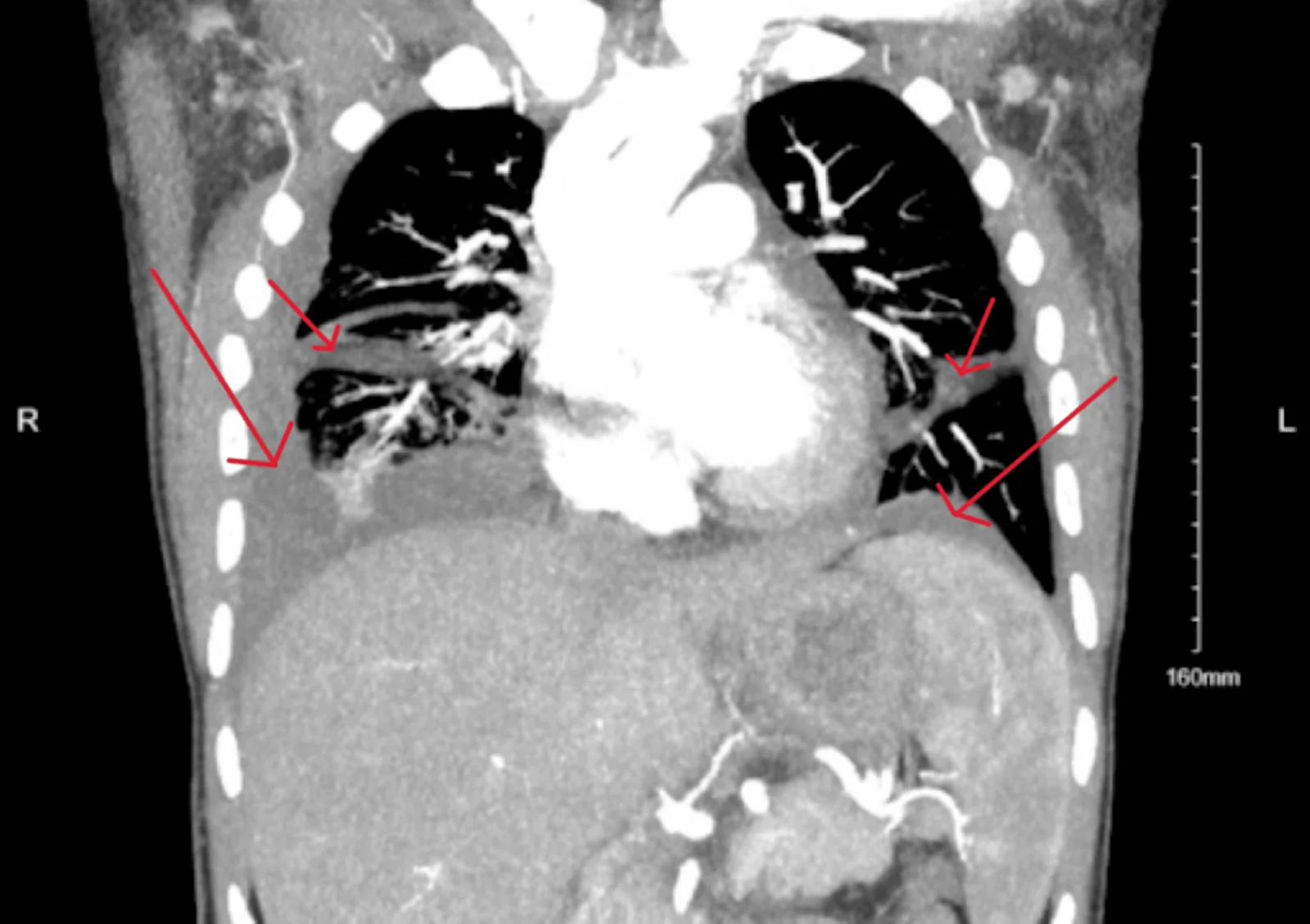
Contrast-enhanced CT of the chest demonstrating bilateral pleural effusions (red arrows) with associated compressive atelectasis of the adjacent lower lobes

**Figure 2:**
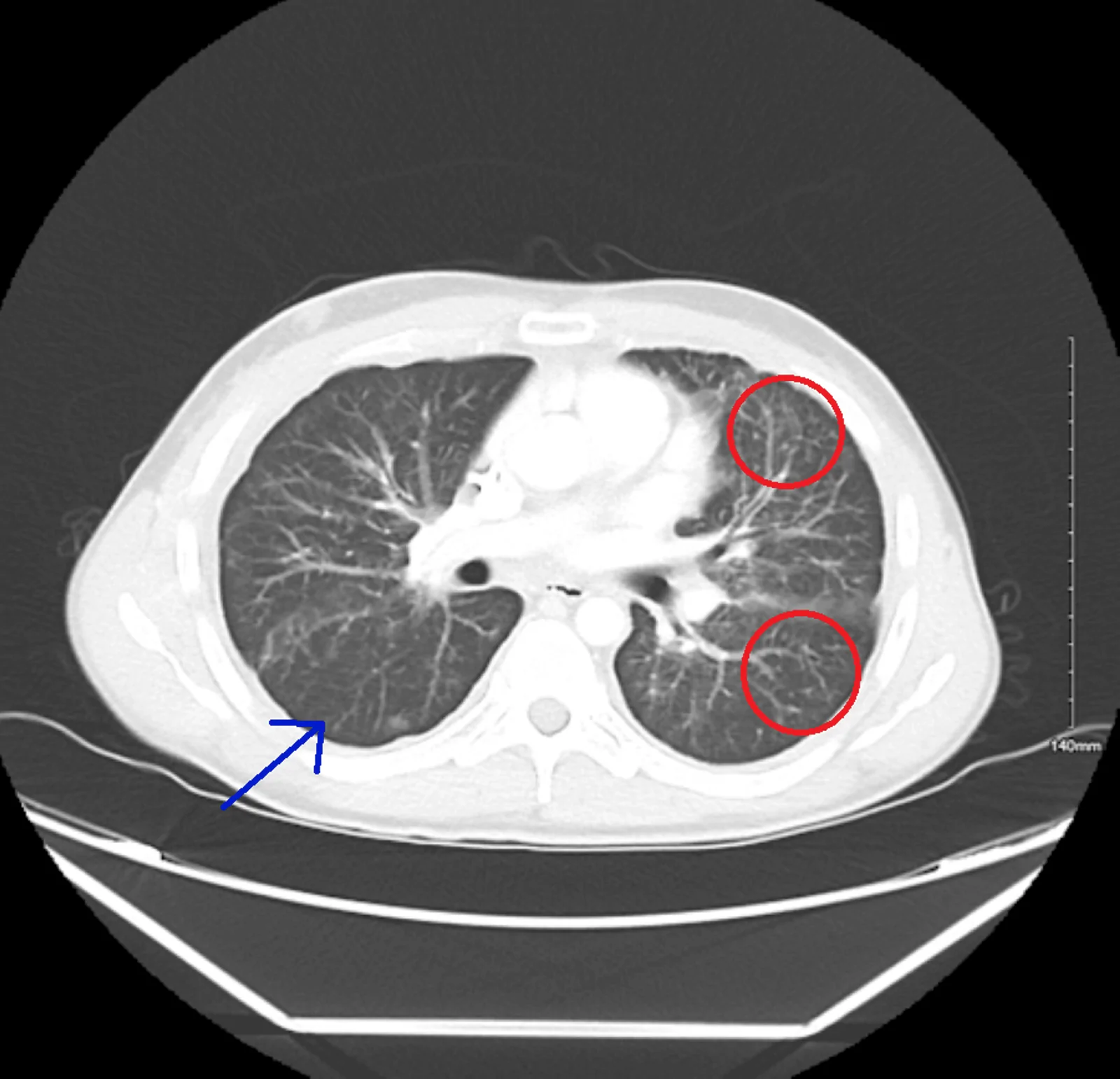
Contrast-enhanced CT of the chest demonstrating diffuse bilateral interstitial infiltrates with associated tree-in-bud opacities (red circles) and basilar parenchymal involvement (blue arrow)

Due to this imaging, the patient was admitted to airborne isolation, and TB was ruled out with three negative sputum samples and a negative *Mycobacterium tuberculosis *PCR. Echocardiography showed normal structures with a normal ejection fraction and a small pericardial effusion. Given the patient’s previously biopsy-confirmed pulmonary involvement of Kaposi sarcoma from prior bronchoscopy with transbronchial biopsy, the imaging findings during this admission were considered most consistent with persistent pulmonary KS, and additional invasive pulmonary diagnostic procedures were deferred. During hospitalization, the patient tested positive for rapid plasma reagin at a 1:4 dilution. The patient reported a previous diagnosis of syphilis, which was treated with penicillin. Infectious disease was consulted and recommended one dose of penicillin and monitoring.

A dermatology consultation was obtained, and a punch biopsy of a skin lesion on the left thigh was performed. Histopathologic examination revealed a spindle cell lesion in the dermis with mild to moderate cytologic atypia, forming irregular, slit-like vascular channels with extravasated red blood cells (Figure 3). Immunohistochemical staining showed that the lesional cells were positive for CD31, CD34, and HHV-8 and negative for AE1/AE3. These findings were consistent with Kaposi sarcoma.

**Figure 3:**
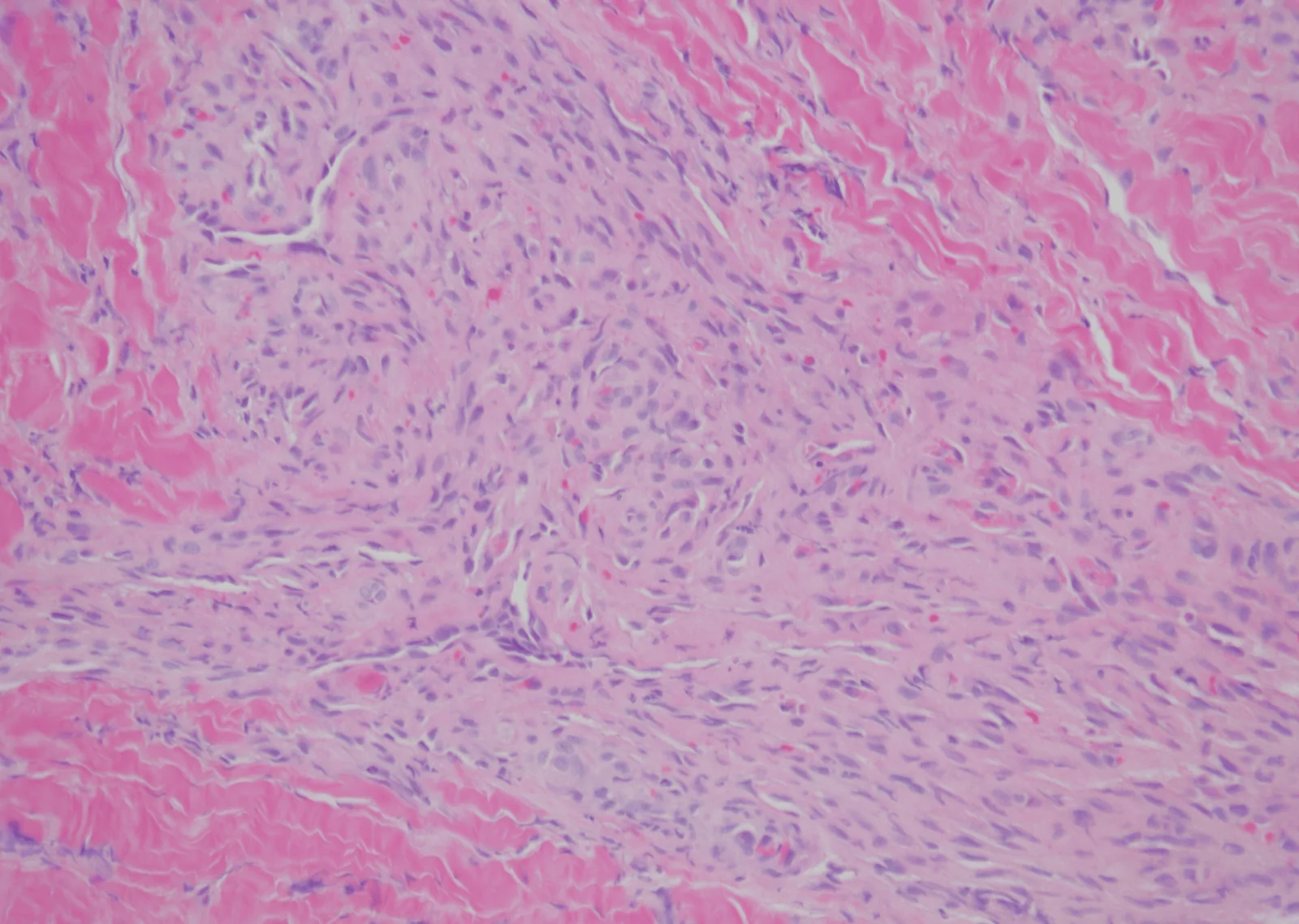
Skin biopsy from the left thigh showing dermal spindle cells forming irregular, slit-like vascular channels with extravasated red blood cells

The patient’s left lower extremity edema improved with leg pumping exercises and physical therapy. He remained asymptomatic with stable vital signs and was discharged with appropriate medical follow-up. Following his discharge, he completed a second round of six doses of PLD. Post-treatment, the patient has repeatedly presented to the ED with a similar presentation of lower extremity edema, limiting his ability to walk. He was then started on pembrolizumab, a programmed cell death protein-1 (PD-1) inhibitor, as escalation therapy for refractory Kaposi sarcoma following progression despite prior treatment.

The patient is currently being followed by hematology-oncology for management of his lower extremity edema and continues to follow with infectious disease for ongoing antiretroviral therapy (ART) management and monitoring of adherence. The edema was thought to be most consistent with lymphatic obstruction related to extensive Kaposi sarcoma involvement, a known complication of advanced KS. Alternative etiologies such as deep vein thrombosis, cardiac dysfunction, and infectious causes were considered, but lower extremity Doppler studies were negative for thrombosis, and echocardiography demonstrated preserved cardiac function. The patient was advised to perform leg pumping exercises while lying or seated to help alleviate the edema. During outpatient follow-up six months later, he was noted to have worsening edema and progressive nodular changes on contrast-enhanced CT of the chest and abdomen, along with diffuse involvement throughout the body (Figure 4). These findings were concerning for progressive KS, prompting initiation of a third course of PLD while continuing pembrolizumab. PLD was selected given its prior clinical benefit and established efficacy in the management of KS.

**Figure 4:**
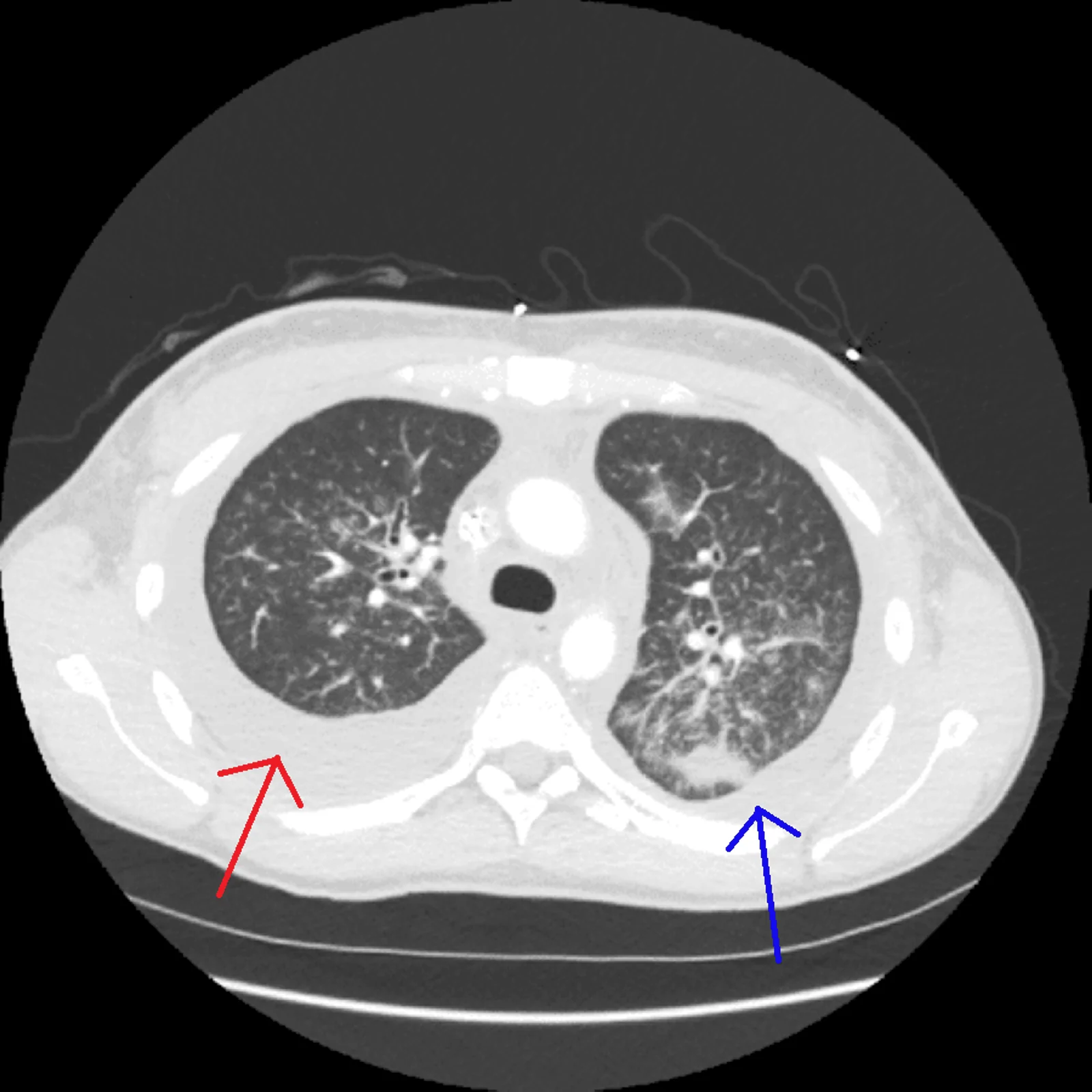
Contrast-enhanced CT of the chest demonstrating bilateral pleural effusions and diffuse bilateral interstitial infiltrates, more pronounced in the right lung (red arrow) compared to the left (blue arrow)

## Discussion

This case highlights the clinical course and management considerations of AIDS-related Kaposi sarcoma (KS). KS is associated with infection by human herpesvirus-8 (HHV-8) and is considered an AIDS-defining illness, notably in patients with a CD4 count <200 cells/mm³. HHV-8-driven angiogenesis, mediated by vascular endothelial growth factor (VEGF) and interleukin-6 (IL-6), contributes to the vascular and hemorrhagic nature of KS lesions [6]. In our patient with refractory AIDS-related Kaposi sarcoma, proliferative cutaneous lesions caused lymphatic obstruction leading to lower extremity edema, while pulmonary involvement most likely explained the bilateral pleural effusions and interstitial changes seen on imaging.

First-line prevention of KS is compliance with ART. Notably, an undetectable HIV viral load is correlated with higher KS remission than with the change in CD4 count [9]. While ART alone can sometimes induce regression of KS by restoring immune function, systemic chemotherapy is generally required in patients with advanced or symptomatic disease, as was the case here.

Adherence to antiretroviral therapy (ART) is the cornerstone of management of AIDS-related Kaposi sarcoma and is associated with improved disease control and higher rates of remission. Notably, viral suppression has been shown to be a stronger predictor of Kaposi sarcoma remission than CD4 count alone [9]. While ART alone can sometimes induce regression of KS by restoring immune function, systemic chemotherapy is generally required in patients with advanced or symptomatic disease, as was the case here.

In a randomized phase III trial involving 258 patients with advanced AIDS-related KS, 133 received pegylated liposomal doxorubicin (PLD) and 125 received doxorubicin, bleomycin, and vincristine (ABV). Both regimens were administered every 14 days for six cycles. Among patients receiving PLD, 45.9% achieved a partial or complete response, compared with 24.8% of patients receiving ABV who achieved a partial response. This difference was statistically significant (p < 0.001) [10].

Paclitaxel, a taxane-based chemotherapeutic agent, is another systemic treatment option. A randomized trial involving 73 analyzable patients found no statistically significant differences in response rate, progression-free survival, or two-year overall survival between paclitaxel and PLD. Both treatments improved pain and swelling [11].

For patients who had already received PLD or paclitaxel for first-line therapy, pembrolizumab is used as second-line therapy for rapidly progressing diseases. The mechanism of action of this medication is by blocking the programmed cell death protein-1 (PD-1) pathway. In a study of 32 patients, pembrolizumab demonstrated an overall response rate of 62% and a median progression-free survival of approximately 28 months [12].

Other investigational agents such as nivolumab, lenalidomide, and bevacizumab are also being studied, reflecting ongoing efforts to target angiogenesis and immune dysregulation in KS [13]. Immune checkpoint inhibitors such as pembrolizumab have therefore opened a new frontier in KS therapy, particularly for refractory cases [14].

Central to the management of AIDS-related Kaposi sarcoma is sustained adherence to antiretroviral therapy (ART). Despite the availability of well-tolerated, once-daily regimens, challenges with adherence and engagement in care persist. Barriers include HIV-related stigma, comorbid mental health conditions, and substance use, all of which can negatively impact treatment adherence. Addressing these factors through a patient-centered approach is essential to support adherence and optimize clinical outcomes [15].

The management of AIDS-related KS also requires a multidisciplinary team approach involving oncology, infectious disease, pulmonology, gastroenterology, dermatology, and social work, with additional emphasis on nutritional support and rehabilitation/exercise in patients with mobility-limiting edema and systemic illness.

## Conclusions

This case highlights the aggressive progression of AIDS-related Kaposi sarcoma in the context of advanced HIV, despite the administration of antiretroviral therapy and standard chemotherapy. Although pegylated liposomal doxorubicin is the recommended first-line treatment, recurrent and progressive disease often necessitates the consideration of alternative therapies, such as pembrolizumab. Continuous clinical monitoring, individualized management, and strict adherence to treatment protocols are crucial for improving outcomes in this patient population.
